# Supplementary material for: Acylation of glycerolipids in mycobacteria
Source: Nat Commun. 2023 Oct 23;14:6694. doi: 10.1038/s41467-023-42478-x (PMC10593935; doi:10.1038/s41467-023-42478-x)

Supplementary Information

Acylation of Glycerolipids in Mycobacteria

Shiva Kumar Angala[1], Ana Carreras-Gonzalez[2,3], Emilie Huc-Claustre[1], Itxaso Anso[4], Devinder Kaur[1¶], Victoria Jones[1], Zuzana Palčeková[1], Juan M. Belardinelli[1], Célia de Sousa-d'Auria[5], Libin Shi[1], Nawel Slama[6], Christine Houssin[5], Annaïk Quémard[6], Michael McNeil[1], Marcelo E. Guerin[2,3,4,7¶¶], and Mary Jackson[1*]

From the [1]Mycobacteria Research Laboratories, Department of Microbiology, Immunology and Pathology, Colorado State University, Fort Collins, CO 80523-1682; [2]Unidad de Biofisica, Centro Mixto Consejo Superior de Investigaciones Cientificas - Universidad del País Vasco/Euskal Herriko Unibertsitatea (CSIC-UPV/EHU), Barrio Sarriena s/n, Leioa, Bizkaia, 48940, Spain; [3]Departamento de Bioquímica, Universidad del País Vasco, Spain; [4]Structural Glycobiology Laboratory, Biocruces Bizkaia Health Research Institute, Cruces University Hospital, Bizkaia 48903; [5]Université Paris-Saclay, CEA, CNRS, Institute for Integrative Biology of the Cell (I2BC), Gif-sur-Yvette, France; [6]Institut de Pharmacologie et de Biologie Structurale (IPBS), CNRS, UPS, Université Toulouse III - Paul Sabatier, Toulouse, France; [7]IKERBASQUE, Basque Foundation for Science, 48009, Bilbao, Spain.

[*]Corresponding author: Mary Jackson; Mary.Jackson@colostate.edu;

[¶]Present address: New England Newborn Screening Program; University of Massachusetts Medical School, Worcester, MA 01605, USA.
[¶¶]Present address: Structural Glycobiology Laboratory, Department of Structural and Molecular Biology; Molecular Biology Institute of Barcelona (IBMB), Spanish National Research Council (CSIC), Barcelona Science Park, c/Baldiri Reixac 4-8, Tower R, 08028 Barcelona, Catalonia, Spain.

Table of contents

Supplementary Table 1: The PlsB and PlsC homologs of *Mtb* H37Rv.

Supplementary Table 2: Effect of silencing *plsM* on the fatty acid composition of *Msmg*.

Supplementary Table 3: MIC of various antibiotics against WT *Msmg* and *Msmg*Δ*plsM*/pMVGH1-$plsC_{coli}$.

Supplementary Table 4: Molecular species of LPA and PA found in the PlsMsmg and PlsB2smg enzymatic products.

Figure S1: Quantitative reverse-transcription PCR showing the level of expression of *plsCcoli* and *plsCsubtilis* in two independent *Msmg* pMVGH1-*plsCcoli* and pMVGH1-*plsCsubtilis* transformants.

Figure S2: Glycerolipid composition of WT *Msmg* mc^{2}155 and *Msmg*Δ*plsM*/pMVGH1-$plsC_{coli.}$

Figure S3: Sliding motility and biofilm-forming capacity of the *Msmg plsM* knock-out mutant rescued with *plsCcoli*.

Figure S4: Purification of recombinant, hexahistidine-tagged, forms PlsMsmg and PlsB2smg from *E. coli* BL21(DE3).

Figure S5: Characterization of the G3P acyltransferase activity of PlsMsmg *in vitro*.

Figure S6: Extracted ion chromatograms (EICs) showing the lysophosphatidic acid (LPA) products resulting from the incubation of membranes prepared from *Msmg*/pMVGH1 and *Msmg*Δ*plsM*/pMVGH1-$plsC_{coli}$ with [^{13}C]-G3P and C18:1-CoA.

Figure S7: LC-MS analysis of the products resulting from PlsB2smg acyltransferase assays using *sn*-1-LPA-$C_{16:0}$ and *sn*-1-LPA-$C_{18:0}$ as acceptor substrates and C18:1-CoA as the acyl donor.

Figure S8: A model for PlsMtb and PlsB2tb substrates recognition.

Supplementary File 1: Lipidomics Reporting Checklist for LC/MS analyses

Supplementary Table 1: The PlsB and PlsC homologs of *Mtb* H37Rv.

Genetic organization of *plsM* and *plsB2* in the genome of *Mycobacterium tuberculosis* H37Rv: *plsM* (in red) [*Rv2182c*] maps adjacent to three mannosyltransferase genes involved in the biosynthesis of phosphatidylinositol mannosides (PIM) and glycosylated derivatives, lipomannan and lipoarabinomannan, including *pimB'* [*Rv2188c*], *mptA* [*Rv2174*] and *mptC* [*Rv2181c*] (in green). *plsB2* (in red) maps adjacent to a putative *plsC* gene [*Rv2483c*] predicted to encode a bifunctional LPA acyltransferase/phosphoserine phosphohydrolase and another acyltransferase gene [*Rv2484c*] whose product was shown to display triacylglycerol synthase activity *in vitro* (both in blue) [Daniel *et al*., 2004].

*Despite its primary sequence homology to bacterial PlsCs, Rv0502 actually lacks the conserved catalytic motif of glycerolipid acyltransferases and is thus unlikely to be functional.

Mtb H37Rv	*Msmg*	*M. leprae*	Homology to bacterial PlsC and PlsB enzymes
**PlsC homologs**<br>**1-acylglycerol-3-phosphate *O*-acyltransferase [EC 2.3.1.51] – Gene Ontology GO 0003841**			
**PlsM**<br>(Rv2182c)<br>(247 aa)	MSMEG_4248<br>(241 aa)	ML0892<br>(245 aa)	25% ID; 50% similarity on a 175 aa overlap to *B. subtilis* YhdO (199 aa)

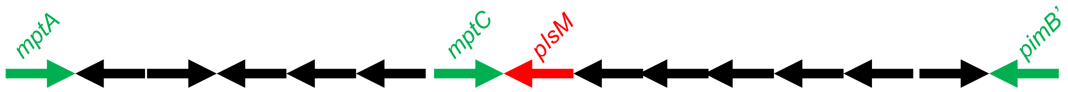

PlsC (Rv2483c)<br>(580 aa)	MSMEG_4704<br>(508 aa)	ML1245<br>(580 aa)	28% ID; 42% similarity on a 167 aa overlap to *E. coli* PlsC (245 aa)
Rv3814c<br>(261 aa)	No ortholog	No ortholog	27% ID; 49% similarity on a 118 aa overlap to *B. subtilis* YhdO (PlsCsubtilis) (199 aa)
Rv3815c<br>(251 aa)	MSMEG_6408<br>(253 aa)	ML0088<br>(pseudogene)	29% ID; 49% similarity on a 141 aa overlap to *B. subtilis* YhdO (PlsCsubtilis) (199 aa)
Rv3816c<br>(259 aa)	MSMEG_6409<br>(263 aa)	ML0087<br>(258 aa)	28% ID; 50% similarity on a 194 aa overlap to *B. subtilis* YhdO (PlsCsubtilis) (199 aa)
Rv3026c<br>(304 aa)	MSMEG_2356<br>(276 aa)	ML1709<br>(pseudogene)	29% ID; 48% similarity on a 164 aa overlap to *N. meningitidis* NlaA (PlsC) (255 aa)
Rv0502*<br>(358 aa)	MSMEG_0947<br>(354 aa)	ML2427c<br>(371 aa)	25% ID; 40% similarity on a 142 aa overlap to *B. subtilis* YhdO (PlsCsubtilis) (199 aa)
**PlsB homologs**<br>**Glycerol-3-phosphate 1-*O*-acyltransferase [EC 2.3.1.15] – Gene Ontology GO 0004366**			
PlsB1 (Rv1551)<br>(621 aa)	No ortholog	No ortholog	29% ID; 44% similarity on a 324 aa overlap to *E. coli* PlsB (827 aa)
**PlsB2**<br>(Rv2482c)<br>(789 aa)	MSMEG_4703<br>(786 aa)	ML1246<br>(776 aa)	27% ID; 47% similarity on a 402 aa overlap to *E. coli* PlsB (827 aa)

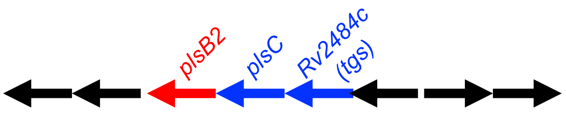

Reference: Daniel, J., Deb, C., Dubey, V. S., Sirakova, T. D., Abomoelak, B., Morbidoni, H. R., and Kolattukudy, P. E. (2004) Induction of a novel class of diacylglycerol acyltransferases and triacylglycerol accumulation in *Mycobacterium tuberculosis* as it goes into a dormancy-like state in culture. *J. Bacteriol.* **186**, 5017-5030

Supplementary Table 2: Effect of silencing *plsM* on the fatty acid composition of *Msmg*.
GC/MS-based determination of fatty acid methyl ester compositions (expressed as percentages) of *Msmg*Δ*plsM*/pSETetR-*plsMsmg* and the *Msmg* control strain harboring an empty pSETetR plasmid grown as in Fig. 1F in 7H9-OADC-Tyloxapol at 37°C to the same OD_{600} (~ 0.5-0.6) in the presence of 1, 5 and 50 ng/mL of ATc. Marked changes in fatty acid profiles are indicated in bold type. The results are means ± SD of duplicate cultures and are representative of two independent experiments using conditional mutant strains *Msmg*Δ*plsM*/pSETetR-*plsMsmg* and *Msmg*Δ*plsM*/pSETetR-*plsMtb.* C19 (TBSA), tuberculostearic acid.

Strain	*Msmg*/pSETetR			*Msmg*Δ*plsM*/pSETetR-*plsMsmg*		
	1 ng/mL	5 ng/mL	50 ng/mL	1 ng/mL	5 ng/mL	50 ng/mL
C14:0	1 ± 0.03	1 ± 0.01	1 ± 0.04	1 ± 0.04	1 ± 0.08	1 ± 0.77
C16:0	27 ± 0.15	27 ±0.31	26 ± 0.02	**22 ± 0.14**	**28 ± 0.07**	**27 ± 0.50**
C18:0	3 ± 0.05	3 ± 0.01	4 ± 0.007	4 ± 0.02	2 ± 0.02	3 ± 0.12
C18:1	37 ± 0.41	36 ± 0.39	34 ± 0.02	**41 ± 0.14**	**32 ± 0.07**	**31 ± 0.50**
C19 (TBSA)	20 ±0.35	19 ± 0.05	21 ± 0.03	**12 ± 0.05**	**29 ± 0.28**	**28 ± 1.37**
C20:0	1 ± 0.08	1 ± 0.05	1 ± 0.02	1 ± 0.04	0.4 ± 0.01	1 ± 0.16
C22:0	2 ± 0.03	2 ± 0.02	3 ± 0.006	3 ± 0.04	1 ± 0.009	2 ± 0.29
C24:0	9 ± 0.03	9 ± 0.23	10 ± 0.28	**15 ± 0.16**	**6 ± 0.001**	**8 ± 1.13**

Supplementary Table 3: MIC of various antibiotics against WT *Msmg* and *Msmg*Δ*plsM*/pMVGH1-*plsC*$_{coli}$.
MIC values are in μg/ml. INH, isoniazid; EMB, ethambutol; STR, streptomycin; CIP, ciprofloxacin; RIF, rifampicin; AMP, ampicillin; CHL, chloramphenicol. MIC determinations were performed twice on two independent culture batches and on two independent *Msmg*Δ*plsM/pMVGH1-plsCcoli* clones with the same results.

**Strain**	**INH**	**EMB**	**STR**	**CIP**	**RIF**	**AMP**	**CHL**
mc^2155 WT	8	0.4	0.2	0.2	20	250	31
*Msmg*Δ*plsM*/pMVGH1-*plsC*$_{coli}$ (clone # 32)	8	0.8	0.4	0.2	40	250	31
*Msmg*Δ*plsM*/pMVGH1-*plsC*$_{coli}$ (clone # 34)	8	0.8	0.2	0.2	40	250	62

<u>Supplementary Table 4</u>: Molecular species of LPA and PA found in the PlsMsmg and PlsB2smg enzymatic products.

	**Enzymatic Products**	**Charge**	**Theoretical mass (*m/z*)**	**Observed mass (*m/z*)**	**Mass error (Da)**	**Mass accuracy (ppm)**
Synthetic standards	*sn*-1 LPA (C16:0)	$[M-H^+]^-$	409.2361	409.2397	0.00360	8.80
	sn-1 LPA (C18:0)	$[M-H^+]^-$	437.2675	437.2701	0.00260	5.95
	sn-1 LPA (C18:1)	$[M-H^+]^-$	435.2518	435.2553	0.00350	8.04
PlsMsmg enzymatic products	*sn*-1 LPA (C16:0)	$[M-H^+]^-$	409.2361	409.2396	0.00350	8.55
	sn-2 LPA (C16:0)	$[M-H^+]^-$	409.2361	409.2400	0.00390	9.53
	sn-1 LPA (C18:0)	$[M-H^+]^-$	437.2675	437.2711	0.00360	8.23
	sn-2 LPA (C18:0)	$[M-H^+]^-$	437.2675	437.2708	0.00330	7.55
	sn-1 LPA (C18:1)	$[M-H^+]^-$	435.2518	435.2558	0.00400	9.19
	sn-2 LPA (C18:1)	$[M-H^+]^-$	435.2518	435.2549	0.00310	7.12
	PA (C16:0/C16:0)	$[M-H^+]^-$	647.4657	647.4717	0.00600	9.27
	PA (C18:0/C18:0)	$[M-H^+]^-$	703.5283	703.5341	0.00580	8.24
	PA (C18:1/C18:1)	$[M-H^+]^-$	699.4970	699.4952	-0.00180	-2.57
	PA (C16:0/C18:0)	$[M-H^+]^-$	675.4970	675.4967	-0.00030	-0.44
	PA (C16:0/C18:1)	$[M-H^+]^-$	673.4814	673.4848	0.00340	5.05
	PA (C18:0/C18:1)	$[M-H^+]^-$	701.5127	701.5156	0.00293	4.18
PlsB2smg enzymatic products	*sn*-1 LPA (C16:0)	$[M-H^+]^-$	409.2361	409.2386	0.00250	6.11
	sn-1 LPA (C18:0)	$[M-H^+]^-$	437.2675	437.2718	0.00430	9.83
	sn-1 LPA (C18:1)	$[M-H^+]^-$	435.2518	435.2561	0.00430	9.88
	PA (C16:0/C16:0)	$[M-H^+]^-$	647.4657	647.4731	0.00740	11.43
	PA (C18:0/C18:0)	$[M-H^+]^-$	703.5283	703.5359	0.00760	10.80
	PA (C18:1/C18:1)	$[M-H^+]^-$	699.4970	699.5045	0.00750	10.72

__Supplementary Figure 1__: **Quantitative reverse-transcription PCR showing the level of expression of *plsCcoli* (green) and *plsCsubtilis* (orange) in two independent *Msmg*/pMVGH1-*plsCcoli* and *Msmg*/pMVGH1-*plsCsubtilis* transformants.**

Transformants were grown to OD_{600} ~ 0.2 in 7H9-ADC-tyloxapol. *plsCcoli* and *plsCsubtilis* cDNA was normalized internally to the *sigA* cDNA in the same sample. Ratios of *plsCcoli*/*sigA* and *plsCsubtilis*/*sigA* mRNA are means ± SD (n = 3 RNA extractions and RT-qPCR reactions). Source data are provided as a Source Data file.

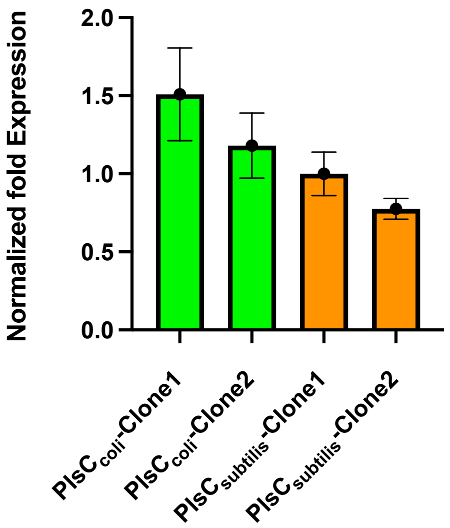

<u>Supplementary Figure 2</u>: Glycerolipid composition of WT *Msmg* mc^{2}155 and *Msmg*Δ*plsM*/pMVGH1-*plsC*$_{coli}$.
(a) Relative abundance of glycerolipids in WT *Msmg* mc^{2}155 and *Msmg*Δ*plsM*/pMVGH1-*plsC*$_{coli}$ (clone # 32) grown in 7H9-ADC-tyloxapol at 37°C to an OD600 nm of ~ 0.8 (see growth curves in Fig. 2B). **(b)** Relative abundance (in percentages) of saturated and unsaturated species within each glycerolipid category (DAG, TAG, PE, CL, PI, Ac_1PIM_2 and Ac_2PIM_2) in WT *Msmg* mc^{2}155 and *Msmg*Δ*plsM*/pMVGH1-*plsC*$_{coli}$ (clone # 32). The results shown in **(a)** and **(b)** are representative of two independent *Msmg*Δ*plsM*/pMVGH1-*plsC*$_{coli}$. clones.
DAG, diacylglycerol; TAG, triacylglycerol; FFA, free fatty acids; PA, phosphatidic acid; PS, phosphatidylserine; PE, phosphatidylethanolamine; PG, phosphatidylglycerol; CL, cardiolipin; PI, phosphatidyl-*myo*-inositol; Ac_1PIM_2, tri-acylated forms of phosphatidyl-*myo*-inositol dimannosides; Ac_2PIM_2, tetra-acylated forms of phosphatidyl-*myo*-inositol dimannosides. Source data are provided as a Source Data file.

a

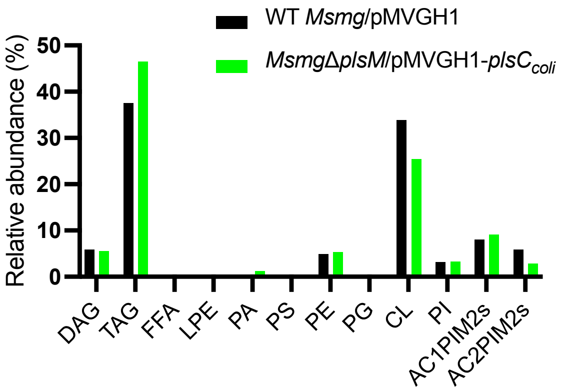

b

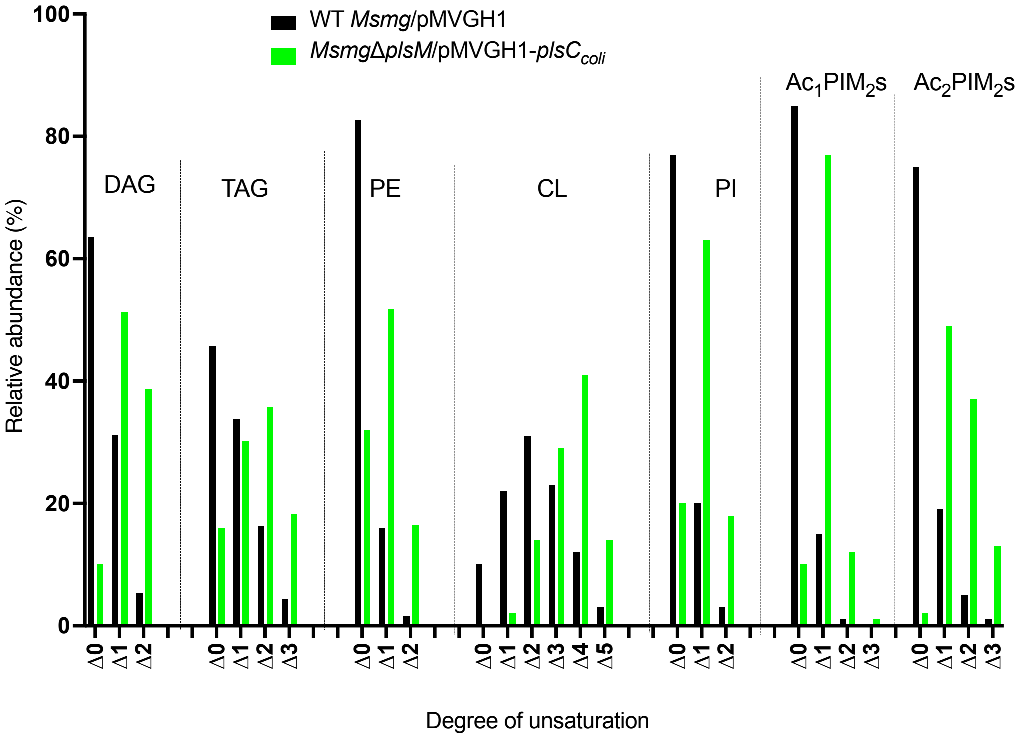

<u>Supplementary Figure 3</u>: Sliding motility and biofilm-forming capacity of the *Msmg plsM* knock-out mutant rescued with *plsCcoli*.

(a) Sliding motility of WT *Msmg* mc^{2}155 and *Msmg*Δ*plsM*/pMVGH1-*plsC*$_{coli}$ (clone # 32) on M63-glucose (Glc) plates with (left pictures) or without (right pictures) added Tween 80. **(b)** Surface pellicle formation in Sauton's medium at 37°C by WT *Msmg* mc^{2}155 (measured after 5 days) and two independent *Msmg*Δ*plsM*/pMVGH1-*plsC*$_{coli}$ clones (# 32 and # 34) measured after 20 days. Despite the longer incubation time, the *plsCcoli* rescued mutants failed to form a thick surface pellicle at the air-liquid interface.

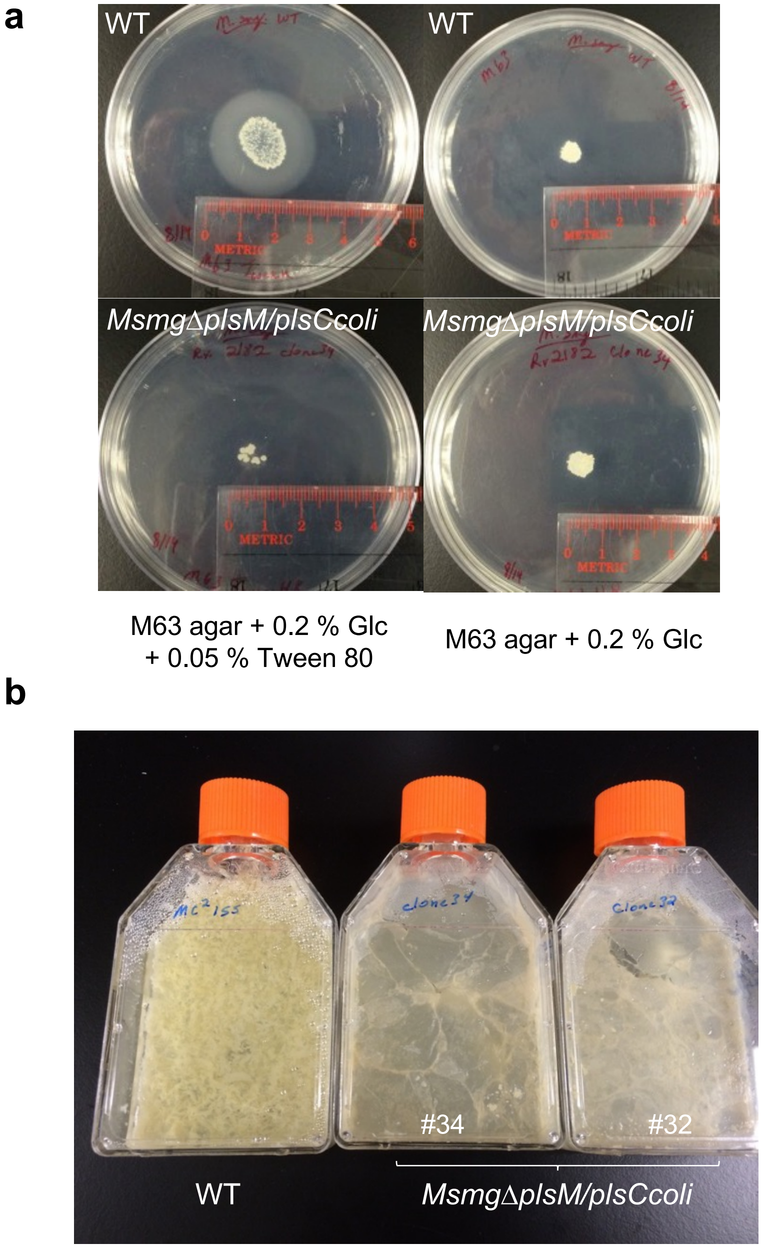

<u>Supplementary Figure 4</u>: Purification of recombinant, hexahistidine-tagged, forms PlsMsmg and PlsB2smg from *E. coli* BL21(DE3).

1 ug (lane 1) and 2 ug (lane 2) of the recombinant PlsMsmg (expected size ~ 27.0 kDa) and PlsB2smg (expected size ~ 88.3 kDa) proteins used in enzyme assays were loaded onto 15% Tris-Glycine-SDS PAGE gels and revealed by Coomassie blue staining.

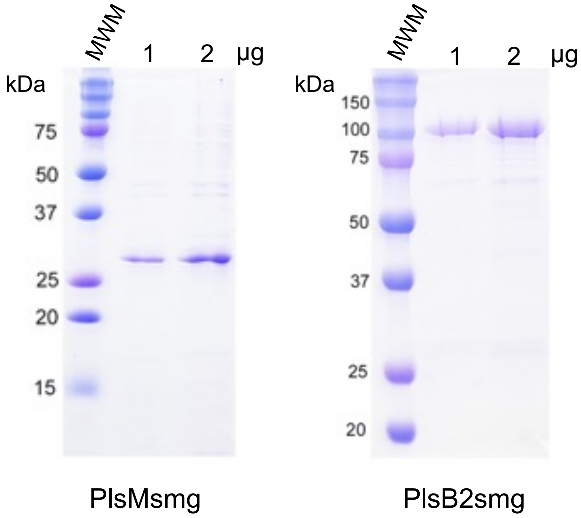

<u>Supplementary Figure 5</u>: Characterization of the G3P acyltransferase activity of PlsMsmg *in vitro*.
(a) Time course. Reactions were carried out as described under Methods with [^{14}C(U)]G3P as the radiolabeled acceptor substrate (20 μM final concentration), 4 μg of purified PlsMsmg and 20 μM C16:0-CoA. The data shown are the means and standard deviations of two assays (n = 2 independent biological samples). **(b)** C16:0-CoA and C16:0-ACP (each used at 2.5 and 10 μM) were compared as acyl donors in reactions containing 20 μg of purified PlsMsmg (or the corresponding mock purification) and 10 μM [^{14}C(U)]G3P. Reactions were run for 30 min. **(c)** Enzyme concentration-dependent formation of LPA (C16:0). Reactions were run for 10 min in the presence of 20 μM [^{14}C(U)]G3P, 20 μM C16:0-CoA and 2, 5 or 20 μg of purified PlsMsmg. The product of the reaction was analyzed by TLC in the solvent system $CHCl_3$:CH_3OH:H_2O (65:25:4 by vol.). The results shown in panels a, b and c are representative of two to three independent experiments. Source data are provided as a Source Data file.

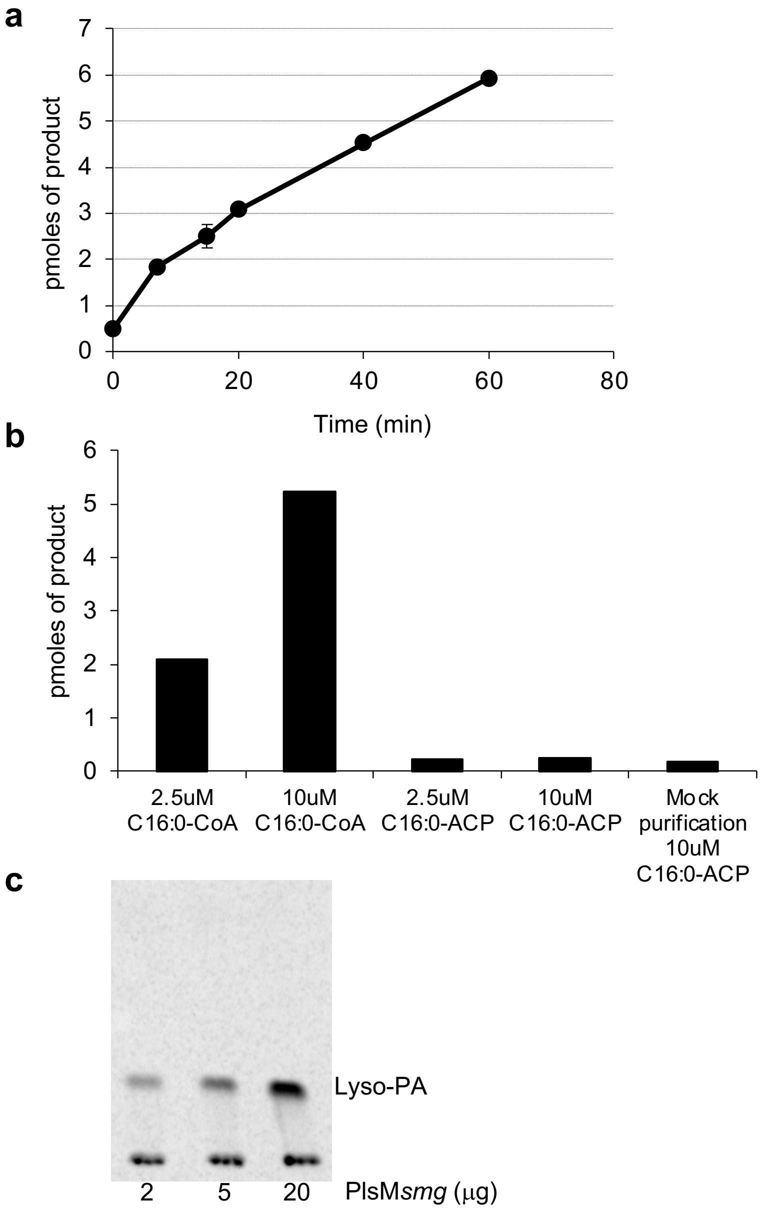

<u>Supplementary Figure 6</u>: Extracted ion chromatograms (EICs) showing the lysophosphatidic acid (LPA) products resulting from the incubation of membranes prepared from *Msmg*/pMVGH1 and *Msmg*Δ*plsM*/pMVGH1-*plsC*$_{coli}$ with [^{13}C]-G3P and C18:1-CoA.

In both membrane preparations, oleyl transfer exclusively occurs at position *sn*-1 of G3P. The results shown are representative of duplicate enzymatic reactions with each membrane preparation.

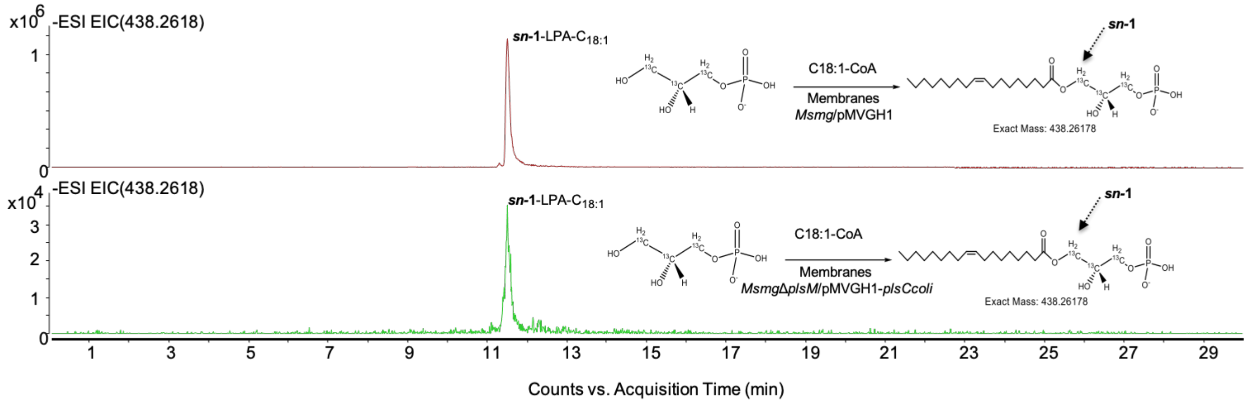

Supplementary Figure 7: LC-MS analysis of the products resulting from PlsB2smg acyltransferase assays using *sn*-1-LPA-$C_{16:0}$ (left panel) and *sn*-1-LPA-$C_{18:0}$ (right panel) and as acceptor substrates and C18:1-CoA as the acyl donor.
The non-enzymatic spontaneous transmigration of the acyl chain from position-1 to position-2 of the *sn*-1-LPA acceptor substrates generates *sn*-2-LPA acceptor substrates (*sn*-2-LPA-$C_{16:0}$ in the left panel and *sn*-1-LPA-$C_{18:0}$ in right panel) that PlsB2smg efficiently uses in the transfer of C18:1 from C18:1-CoA to position *sn*-1 generating 1-oleoyl-2-palmitoyl-*sn*-G3P (left panel) and 1-oleoyl-2-stearoyl-*sn*-G3P (right panel). The position of the oleyl chain in the reaction products was ascertained by enzymatic digestion with phospholipase A2 which yielded, in both cases, the expected *sn*-1-LPA-$C_{18:1}$ products comigrating with the 1-oleoyl-2-hydroxy-G3P commercial standard.

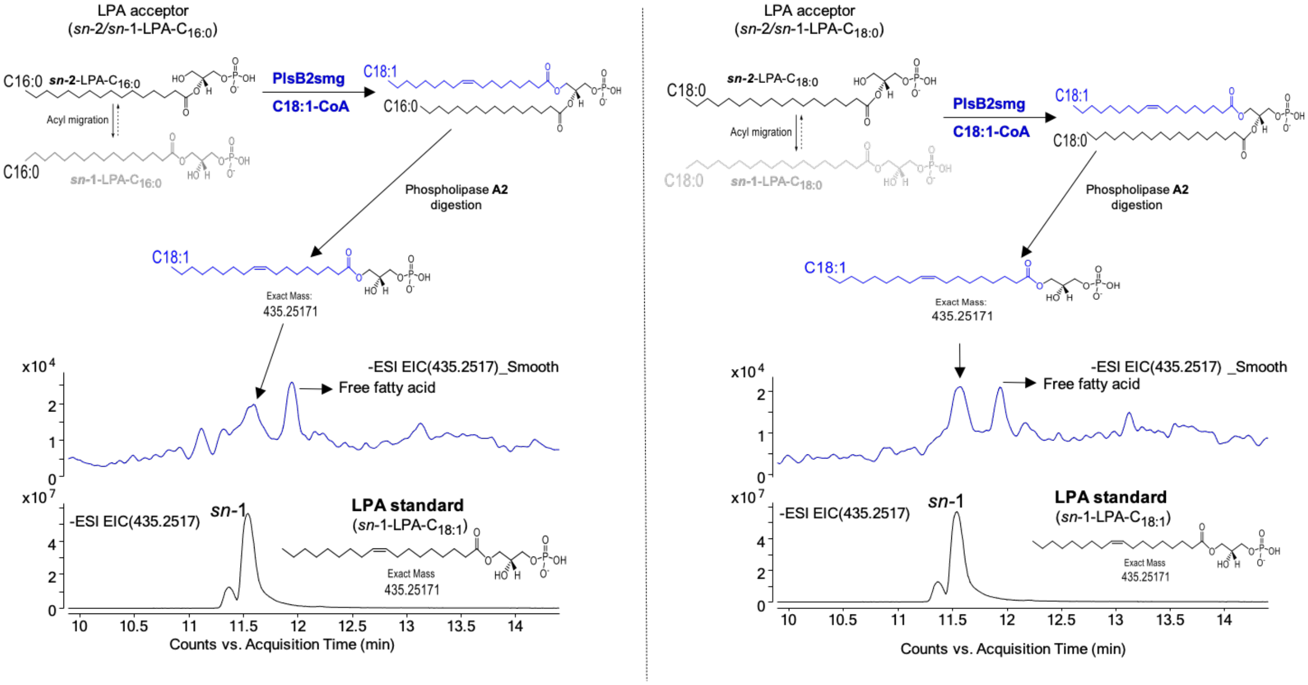

<u>Supplementary Figure 8</u>: A model for PlsMtb and PlsB2tb substrates recognition.

(A) A model for 3D structure prediction of PlsMtb by Alphafold2. The ribbon representation is colored in grey. **(B)** The palmitoyl moiety of C16:0-CoA makes interactions with W2, H41, S47, F48, P51, L52, F60, F80, Y81, S84, Q86 along the groove's walls and with F6, K7 and Y3 in the bottom, supporting PlsMtb's specificity for C16-length donor substrates. The 4-phosphopantetheinate moiety of C16:0-CoA is placed nearby the catalytic residues H41 and D46 and stabilized by Y117 with the adenosine 3′,5′-ADP moiety making interactions with residues R91, T121, R137, T133, R194, Y216 and K132. G3P is stabilized by R122, Y66 and N77 residues (in orange), which perform H bond interactions with the phosphate group of the ligand, facilitating the correct orientation of the OH2 in the catalytic site of the enzyme. **(C)** Cartoon representation of the 3D structure prediction of PlsB2tb by Alphafold2. The predicted N-terminal HAD domain is shown in orange, the acyltransferase domain displaying a α/β architecture is colored in grey, while the C-terminal α helical domain is shown in light blue. **(D)** The stearoyl moiety of C18:0-CoA makes interactions with E290, M289, V233, P286, S317, M314, M319, M299, V236, V283 and Y279 along the groove's walls. The 4-phosphopantetheinate moiety of C18:0-CoA is placed nearby the catalytic residues H276 and D281 and further stabilized by L305, N304 and I322, with the adenosine 3′,5′-ADP moiety making interactions with residues N325, I326, K368, N328, K333, R466 and R324. The phosphate group of G3P is stabilized by the R358 and R360 residues, and T357, facilitating the correct orientation of the OH1 in the catalytic site of the enzyme.

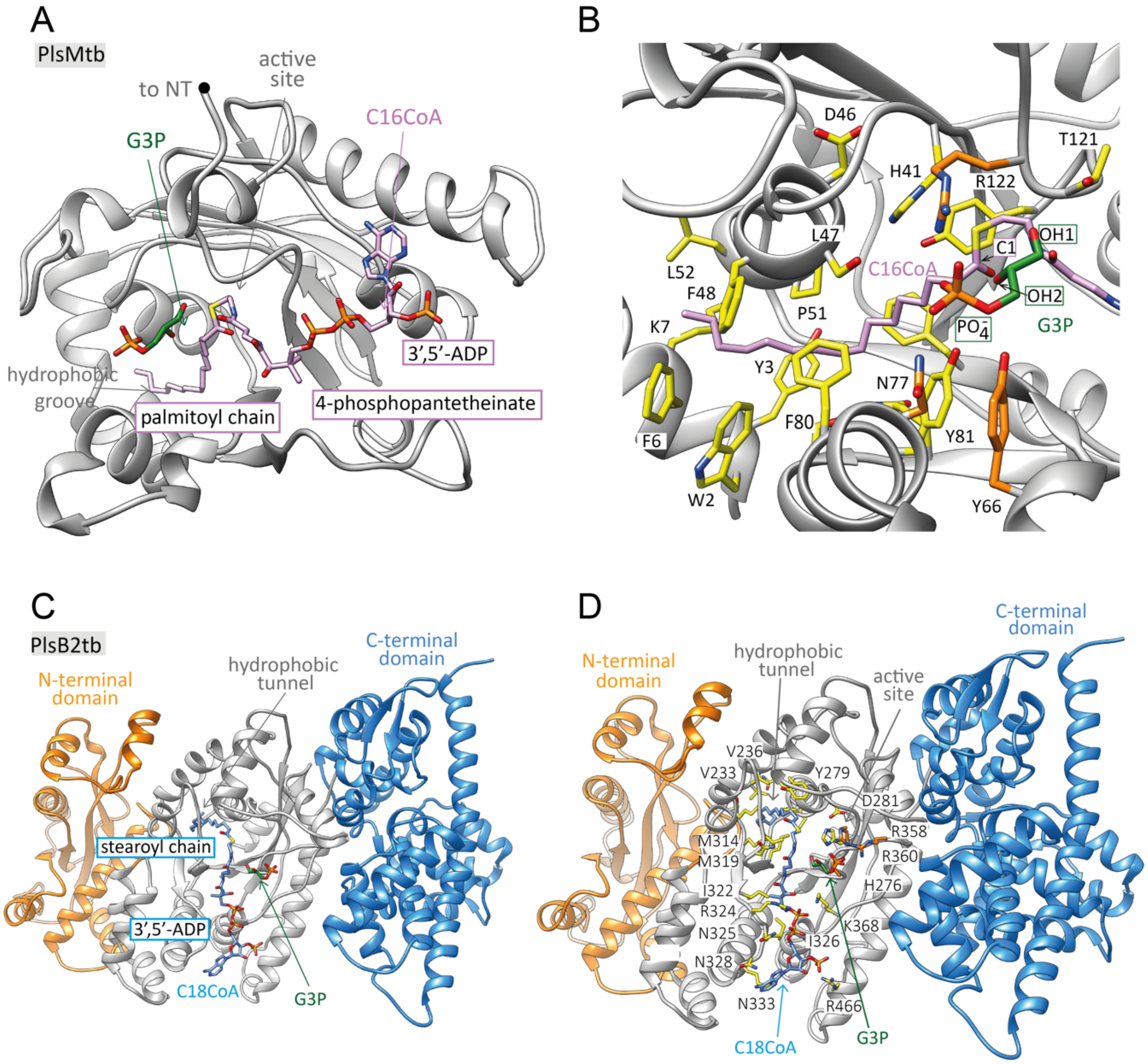

<u>Supplementary File 1</u>: Lipidomics Reporting Checklist for LC/MS analyses

(see next 10 pages)

Separation Workflow

Created by https://lipidomicstandards.org, version v2.2.5

Overall study design

Title of the study	A Unique Pathway to the Acylation of Glycerolipids in Mycobacteria		
Document creation date	09/08/2023	Corresponding Email	mary.jackson@colostate.edu
Principle investigator	Mary Jackson / Shiva Angala-Mary Jackson laboratory	Is the workflow targeted or untargeted?	Untargeted
Institution	Colorado State University	Clinical	No

Lipid extraction

Extraction method	chloroform:methanol (2:1, v/v)	Were internal standards added prior extraction?	No
pH adjustment	None		

Analytical platform

Number of separation dimensions	One dimension	Ion source	ESI
Separation Type 1	LC	MS Level	MS1
Separation Mode 1 (Liquid)	RP	Mass resolution for detected ion at MS1	High resolution
Detector	Mass spectrometer	Resolution at m/z 200 at MS1	20824
MS type	Orbitrap	Mass accuracy in ppm at MS1	5
MS vendor	Thermo	Was/Were additional dimension/techniques used	No

Quality control

Blanks	No	Quality control	No

Method qualification and validation

Method validation	No

Reporting

Are reported raw data uploaded into repository?	No	Raw data upload	No
Are metadata available?	No	Additional comments	-

Lipid Class Descriptions

1) MG[M+NH4]+ / Lipid identification

Lipid class	MG	Check isomer overlap	No
MS Level for identification	MS1	RT verified by standard	Yes
Identification level	Species level	Separation of isobaric/isomeric interferece confirmed	No
Polarity mode	Positive	Model for separation prediction	No
Type of positive (precursor)ion	[M+NH4]+	Additional dimension/techniques	-
Isotope correction at MS1	No	Lipid Identification Software	Homemade
MS1 verified by standard	Yes	Data manipulation	-
Background check at MS1	No	Nomenclature for intact lipid molecule	No
Did you presume assumptions for identification?	No	Further identification remarks	-

1) MG[M+NH4]+ / Lipid quantification

Quantitative	Yes	Limit of quantification	No
MS Level for quantification	MS1	Normalization to reference	No
Internal lipid standard(s) MS1		Lipid Quantification Software	LipidXplorer

Internal standard	Endogenous subclass
SPLASH LIPIDOMIX Mass Spec Standard	MG 18:1-d7

Type of quantification	Internal standard amount	Batch correction	No
Response correction	No	Further quantification remarks	-
Type I isotope correction	No		

2) FA[M-H]- / Lipid identification

Lipid class	FA	Check isomer overlap	No
MS Level for identification	MS1	RT verified by standard	No
Identification level	Molecular species level	Separation of isobaric/isomeric interferece confirmed	No
Polarity mode	Negative	Model for separation prediction	No
Type of negative (precursor)ion	[M-H]-	Additional dimension/techniques	-
Isotope correction at MS1	No	Lipid Identification Software	Homemade
MS1 verified by standard	No	Data manipulation	-
Background check at MS1	No	Nomenclature for intact lipid molecule	No
Did you presume assumptions for identification?	No	Further identification remarks	-

2) FA[M-H]- / Lipid quantification

Quantitative	Yes	Limit of quantification	No
MS Level for quantification	MS1	Normalization to reference	No
Internal lipid standard(s) MS1		Lipid Quantification Software	LipidXplorer

Internal standard	Endogenous subclass
SPLASH LIPIDOMIX Mass Spec Standard	18:1-d7 Lyso PE

Type of quantification	Internal standard amount	Batch correction	No
Response correction	No	Further quantification remarks	Due to the absence of a deuterated FFA internal standard in the SPLASH LIPIDOMIX standard, we used deuterated Lyso PE peak areas for semi-quantifying FFA.
Type I isotope correction	No		

3) DG[M+NH4]+ / Lipid identification

Lipid class	DG	Check isomer overlap	No
MS Level for identification	MS1	RT verified by standard	Yes
Identification level	Molecular species level	Separation of isobaric/isomeric interferece confirmed	No
Polarity mode	Positive	Model for separation prediction	No
Type of positive (precursor)ion	[M+NH4]+	Additional dimension/techniques	-
Isotope correction at MS1	No	Lipid Identification Software	Homemade
MS1 verified by standard	Yes	Data manipulation	-
Background check at MS1	No	Nomenclature for intact lipid molecule	No
Did you presume assumptions for identification?	No	Further identification remarks	-

3) DG[M+NH4]+ / Lipid quantification

Quantitative	Yes	Limit of quantification	No
MS Level for quantification	MS1	Normalization to reference	No
Internal lipid standard(s) MS1		Lipid Quantification Software	Homemade

Internal standard	Endogenous subclass
SPLASH LIPIDOMIX Mass Spec Standard	DG 15:0-18:1(d7)

Type of quantification	Internal standard amount	Batch correction	No
Response correction	No	Further quantification remarks	-
Type I isotope correction	No		

4) TG[M+NH4]+ / Lipid identification

Lipid class	TG	Check isomer overlap	No
MS Level for identification	MS1	RT verified by standard	Yes
Identification level	Molecular species level	Separation of isobaric/isomeric interferece confirmed	No
Polarity mode	Positive	Model for separation prediction	No
Type of positive (precursor)ion	[M+NH4]+	Additional dimension/techniques	-
Isotope correction at MS1	No	Lipid Identification Software	Homemade
MS1 verified by standard	Yes	Data manipulation	-
Background check at MS1	No	Nomenclature for intact lipid molecule	No
Did you presume assumptions for identification?	No	Further identification remarks	-

4) TG[M+NH4]+ / Lipid quantification

Quantitative	Yes	Limit of quantification	No
MS Level for quantification	MS1	Normalization to reference	No
Internal lipid standard(s) MS1		Lipid Quantification Software	Homemade

Internal standard	Endogenous subclass
SPLASH LIPIDOMIX Mass Spec Standard	TG 15:0-18:1 (d7)-15:0

Type of quantification	Internal standard amount	Batch correction	No
Response correction	No	Further quantification remarks	-
Type I isotope correction	No		

5) LPE[M-H]- / Lipid identification

Lipid class	LPE	Check isomer overlap	No
MS Level for identification	MS1	RT verified by standard	Yes
Identification level	Molecular species level	Separation of isobaric/isomeric interferece confirmed	No
Polarity mode	Negative	Model for separation prediction	No
Type of negative (precursor)ion	[M-H]-	Additional dimension/techniques	-
Isotope correction at MS1	No	Lipid Identification Software	Homemade
MS1 verified by standard	Yes	Data manipulation	-
Background check at MS1	No	Nomenclature for intact lipid molecule	No
Did you presume assumptions for identification?	No	Further identification remarks	-

5) LPE[M-H]- / Lipid quantification

Quantitative	Yes	Limit of quantification	No
MS Level for quantification	MS1	Normalization to reference	No
Internal lipid standard(s) MS1		Lipid Quantification Software	Homemade

Internal standard	Endogenous subclass
SPLASH LIPIDOMIX Mass Spec Standard	Lyso PE 18:1(d7)

Type of quantification	Internal standard amount	Batch correction	No
Response correction	No	Further quantification remarks	-
Type I isotope correction	No		

6) PA[M-H]- / Lipid identification

Lipid class	PA	Check isomer overlap	No
MS Level for identification	MS1	RT verified by standard	Yes
Identification level	Molecular species level	Separation of isobaric/isomeric interferece confirmed	No
Polarity mode	Negative	Model for separation prediction	No
Type of negative (precursor)ion	[M-H]-	Additional dimension/techniques	-
Isotope correction at MS1	No	Lipid Identification Software	Homemade
MS1 verified by standard	Yes	Data manipulation	-
Background check at MS1	No	Nomenclature for intact lipid molecule	No
Did you presume assumptions for identification?	No	Further identification remarks	-

6) PA[M-H]- / Lipid quantification

Quantitative	Yes	Limit of quantification	No
MS Level for quantification	MS1	Normalization to reference	No
Internal lipid standard(s) MS1		Lipid Quantification Software	Homemade

Internal standard	Endogenous subclass
SPLASH LIPIDOMIX Mass Spec Standard	PA 15:0-18:1(d7)

Type of quantification	Internal standard amount	Batch correction	No
Response correction	No	Further quantification remarks	-
Type I isotope correction	No		

7) PS[M-H]- / Lipid identification

Lipid class	PS	Check isomer overlap	No
MS Level for identification	MS1	RT verified by standard	Yes
Identification level	Molecular species level	Separation of isobaric/isomeric interferece confirmed	No
Polarity mode	Negative	Model for separation prediction	No
Type of negative (precursor)ion	[M-H]-	Additional dimension/techniques	-
Isotope correction at MS1	No	Lipid Identification Software	Homemade
MS1 verified by standard	Yes	Data manipulation	-
Background check at MS1	No	Nomenclature for intact lipid molecule	No
Did you presume assumptions for identification?	No	Further identification remarks	-

7) PS[M-H]- / Lipid quantification

Quantitative	Yes	Limit of quantification	No
MS Level for quantification	MS1	Normalization to reference	No
Internal lipid standard(s) MS1		Lipid Quantification Software	Homemade

Internal standard	Endogenous subclass
SPLASH LIPIDOMIX Mass Spec Standard	PS 15:0-18:1(d7)

Type of quantification	Internal standard amount	Batch correction	No
Response correction	No	Further quantification remarks	-
Type I isotope correction	No		

8) PG[M-H]- / Lipid identification

Lipid class	PG	Check isomer overlap	No
MS Level for identification	MS1	RT verified by standard	Yes
Identification level	Molecular species level	Separation of isobaric/isomeric interferece confirmed	No
Polarity mode	Negative	Model for separation prediction	No
Type of negative (precursor)ion	[M-H]-	Additional dimension/techniques	-
Isotope correction at MS1	No	Lipid Identification Software	Homemade
MS1 verified by standard	Yes	Data manipulation	-
Background check at MS1	No	Nomenclature for intact lipid molecule	No
Did you presume assumptions for identification?	No	Further identification remarks	-

8) PG[M-H]- / Lipid quantification

Quantitative	Yes	Limit of quantification	No
MS Level for quantification	MS1	Normalization to reference	No
Internal lipid standard(s) MS1		Lipid Quantification Software	Homemade

Internal standard	Endogenous subclass
SPLASH LIPIDOMIX Mass Spec Standard	PG 15:0-18:1(d7)

Type of quantification	Internal standard amount	Batch correction	No
Response correction	No	Further quantification remarks	-
Type I isotope correction	No		

9) PE[M-H]- / Lipid identification

Lipid class	PE	Check isomer overlap	No
MS Level for identification	MS1	RT verified by standard	Yes
Identification level	Molecular species level	Separation of isobaric/isomeric interferece confirmed	No
Polarity mode	Negative	Model for separation prediction	No
Type of negative (precursor)ion	[M-H]-	Additional dimension/techniques	-
Isotope correction at MS1	No	Lipid Identification Software	Homemade
MS1 verified by standard	Yes	Data manipulation	-
Background check at MS1	No	Nomenclature for intact lipid molecule	No
Did you presume assumptions for identification?	No	Further identification remarks	-

9) PE[M-H]- / Lipid quantification

Quantitative	Yes	Limit of quantification	No
MS Level for quantification	MS1	Normalization to reference	No
Internal lipid standard(s) MS1		Lipid Quantification Software	Homemade

Internal standard	Endogenous subclass
SPLASH LIPIDOMIX Mass Spec Standard	PE 15:0-18:1(d7)

Type of quantification	Internal standard amount	Batch correction	No
Response correction	No	Further quantification remarks	-
Type I isotope correction	No		

10) CL[M-H]- / Lipid identification

Lipid class	CL	Check isomer overlap	No
MS Level for identification	MS1	RT verified by standard	Yes
Identification level	Molecular species level	Separation of isobaric/isomeric interferece confirmed	No
Polarity mode	Negative	Model for separation prediction	No
Type of negative (precursor)ion	[M-H]-	Additional dimension/techniques	-
Isotope correction at MS1	No	Lipid Identification Software	Homemade
MS1 verified by standard	Yes	Data manipulation	-
Background check at MS1	No	Nomenclature for intact lipid molecule	No
Did you presume assumptions for identification?	No	Further identification remarks	-

10) CL[M-H]- / Lipid quantification

Quantitative	Yes	Limit of quantification	No
MS Level for quantification	MS1	Normalization to reference	No
Internal lipid standard(s) MS1		Lipid Quantification Software	Homemade
Type of quantification	Internal standard amount	Batch correction	No
Response correction	No	Further quantification remarks	Due to the absence of a deuterated CL internal standard in the SPLASH LIPIDOMIX Mass Spec Standard, we used deuterated PI peak areas for semi-quantifying CL.
Type I isotope correction	No		

Internal lipid standard(s) MS1:

Internal standard	Endogenous subclass
SPLASH LIPIDOMIX Mass Spec Standard	PI 15:0-18:1(d7)

11) PI[M-H]- / Lipid identification

Lipid class	PI	Check isomer overlap	No
MS Level for identification	MS1	RT verified by standard	Yes
Identification level	Molecular species level	Separation of isobaric/isomeric interferece confirmed	No
Polarity mode	Negative	Model for separation prediction	No
Type of negative (precursor)ion	[M-H]-	Additional dimension/techniques	-
Isotope correction at MS1	No	Lipid Identification Software	Homemade
MS1 verified by standard	Yes	Data manipulation	-
Background check at MS1	No	Nomenclature for intact lipid molecule	No
Did you presume assumptions for identification?	No	Further identification remarks	-

11) PI[M-H]- / Lipid quantification

Quantitative	Yes	Limit of quantification	No
MS Level for quantification	MS1	Normalization to reference	No
Internal lipid standard(s) MS1		Lipid Quantification Software	Homemade

Internal standard	Endogenous subclass
SPLASH LIPIDOMIX Mass Spec Standard	PI 15:0-18:1(d7)

Type of quantification	Internal standard amount	Batch correction	No
Response correction	No	Further quantification remarks	-
Type I isotope correction	No		

12) Ac1PIM2[M-H]- / Lipid identification

Lipid class	Ac1PIM2	Check isomer overlap	No
MS Level for identification	MS1	RT verified by standard	No
Identification level	Molecular species level	Separation of isobaric/isomeric interferece confirmed	No
Polarity mode	Negative	Model for separation prediction	No
Type of negative (precursor)ion	[M-H]-	Additional dimension/techniques	-
Isotope correction at MS1	No	Lipid Identification Software	Homemade
MS1 verified by standard	No	Data manipulation	-
Background check at MS1	No	Nomenclature for intact lipid molecule	No
Did you presume assumptions for identification?	No	Further identification remarks	-

12) Ac1PIM2[M-H]- / Lipid quantification

Quantitative	Yes	Limit of quantification	No
MS Level for quantification	MS1	Normalization to reference	No
Internal lipid standard(s) MS1		Lipid Quantification Software	LipidXplorer

Internal standard	Endogenous subclass
SPLASH LIPIDOMIX Mass Spec Standard	PI 15:0-18:1(d7)

Type of quantification	Internal standard amount	Batch correction	No
Response correction	No	Further quantification remarks	Due to the absence of a deuterated Ac1PIM2 internal standard in the SPLASH LIPIDOMIX Mass Spec Standard, we used deuterated PI peak areas for semi-quantifying Ac1PIM2.
Type I isotope correction	No		

13) Ac2PIM2[M-H]- / Lipid identification

Lipid class	Ac2PIM2	Check isomer overlap	No
MS Level for identification	MS1	RT verified by standard	No
Identification level	Molecular species level	Separation of isobaric/isomeric interferece confirmed	No
Polarity mode	Negative	Model for separation prediction	No
Type of negative (precursor)ion	[M-H]-	Additional dimension/techniques	-
Isotope correction at MS1	No	Lipid Identification Software	Homemade
MS1 verified by standard	No	Data manipulation	-
Background check at MS1	No	Nomenclature for intact lipid molecule	No
Did you presume assumptions for identification?	No	Further identification remarks	-

13) Ac2PIM2[M-H]- / Lipid quantification

Quantitative	Yes	Limit of quantification	No
MS Level for quantification	MS1	Normalization to reference	No
Internal lipid standard(s) MS1		Lipid Quantification Software	Homemade
Type of quantification	Internal standard amount	Batch correction	No
Response correction	No	Further quantification remarks	Due to the absence of a deuterated Ac2PIM2 internal standard in the SPLASH LIPIDOMIX Mass Spec Standard, we used deuterated PI peak areas for semi-quantifying Ac2PIM2.
Type I isotope correction	No		

Internal lipid standard(s) MS1:

Internal standard	Endogenous subclass
SPLASH LIPIDOMIX Mass Spec Standard	PI 15:0-18:1(d7)

Figure S5C: Uncropped Image

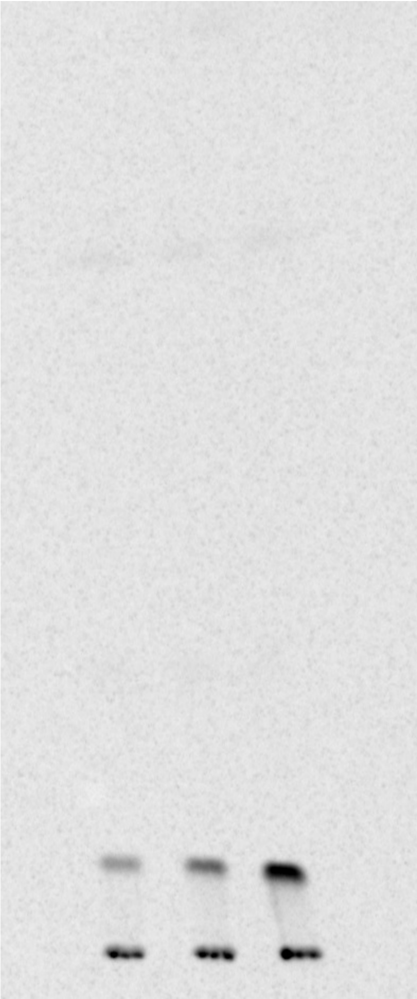

Supplement: Supplementary file 1 — Supplementary Information [file 41467_2023_42478_MOESM1_ESM.pdf]
