# Supplementary material for: Acylation of glycerolipids in mycobacteria
Source: Nat Commun. 2023 Oct 23;14:6694. doi: 10.1038/s41467-023-42478-x (PMC10593935; doi:10.1038/s41467-023-42478-x)
Supplement: Supplementary file 3 — Reporting Summary [file 41467_2023_42478_MOESM3_ESM.pdf]

nature portfolio

Corresponding author(s): Mary Jackson

Last updated by author(s): Oct 2, 2023

Reporting Summary

Nature Portfolio wishes to improve the reproducibility of the work that we publish. This form provides structure for consistency and transparency in reporting. For further information on Nature Portfolio policies, see our Editorial Policies and the Editorial Policy Checklist.

Statistics

For all statistical analyses, confirm that the following items are present in the figure legend, table legend, main text, or Methods section.

n/a	Confirmed	
[ ]	[x]	The exact sample size (*n*) for each experimental group/condition, given as a discrete number and unit of measurement
[ ]	[x]	A statement on whether measurements were taken from distinct samples or whether the same sample was measured repeatedly
[ ]	[x]	The statistical test(s) used AND whether they are one- or two-sided<br>*Only common tests should be described solely by name; describe more complex techniques in the Methods section.*
[x]	[ ]	A description of all covariates tested
[x]	[ ]	A description of any assumptions or corrections, such as tests of normality and adjustment for multiple comparisons
[ ]	[x]	A full description of the statistical parameters including central tendency (e.g. means) or other basic estimates (e.g. regression coefficient) AND variation (e.g. standard deviation) or associated estimates of uncertainty (e.g. confidence intervals)
[ ]	[x]	For null hypothesis testing, the test statistic (e.g. F, t, r) with confidence intervals, effect sizes, degrees of freedom and P value noted<br>*Give P values as exact values whenever suitable.*
[x]	[ ]	For Bayesian analysis, information on the choice of priors and Markov chain Monte Carlo settings
[x]	[ ]	For hierarchical and complex designs, identification of the appropriate level for tests and full reporting of outcomes
[x]	[ ]	Estimates of effect sizes (e.g. Cohen's *d*, Pearson's *r*), indicating how they were calculated

Our web collection on statistics for biologists contains articles on many of the points above.

Software and code

Policy information about availability of computer code

Data collection	LC/MS (Agilent Mass Hunter workstation qualitative analysis version 10.0, build 10.0.10305.0); GC/MS (Varian MS Workstation MS Data Review version 6.9 (service pack 1) and Thermo Scientific TM Dionex TM Chromeleon TM 7 Chromatography Data System Version 7.2.10 (23925)); RT-qPCR (Bio-Rad CFX Manager version 1.6.541.1028).
Data analysis	PerkinElmer ChemDraw version: 21.0.0.28; Prism version 9.5.1; LC/MS data were analyzed using Agilent Mass Hunter workstation qualitative analysis version 10.0, build 10.0.10305.0; GC/MS data were analyzed using Thermo Scientific Chromeleon version 7.2.10; structure model predictions were computationally calculated by Alphafold software. Ligand docking was performed using AutoDock Vina employing standard parameters. Molecular Dynamics simulations were carried out with AMBER 20 package implemented with ff14SB and GLYCAM06 force fields. Structure based homologues search and Z-score values were produced by using DALI and molecular modelling visualization was performed using USCF Chimera.

For manuscripts utilizing custom algorithms or software that are central to the research but not yet described in published literature, software must be made available to editors and reviewers. We strongly encourage code deposition in a community repository (e.g. GitHub). See the Nature Portfolio guidelines for submitting code & software for further information.

Data

Policy information about availability of data

All manuscripts must include a data availability statement. This statement should provide the following information, where applicable:

- Accession codes, unique identifiers, or web links for publicly available datasets
- A description of any restrictions on data availability
- For clinical datasets or third party data, please ensure that the statement adheres to our policy

The data generated in this study, including the processed LC/MS data, are available within the paper, its supplementary information files and Source Data file. Raw LC/MS data are available as Agilent Mass Hunter workstation files from the corresponding author upon reasonable request. Source data are provided with this paper.

Human research participants

Policy information about studies involving human research participants and Sex and Gender in Research.

Reporting on sex and gender	N/A
Population characteristics	N/A
Recruitment	N/A
Ethics oversight	N/A

Note that full information on the approval of the study protocol must also be provided in the manuscript.

Field-specific reporting

Please select the one below that is the best fit for your research. If you are not sure, read the appropriate sections before making your selection.

☒ Life sciences ☐ Behavioural & social sciences ☐ Ecological, evolutionary & environmental sciences

For a reference copy of the document with all sections, see nature.com/documents/nr-reporting-summary-flat.pdf

Life sciences study design

All studies must disclose on these points even when the disclosure is negative.

Sample size	No statistical calculation was performed to determine sample size. Instead, the number of replicates was chosen based on previously published studies (https://pubmed.ncbi.nlm.nih.gov/12851411/; https://pubmed.ncbi.nlm.nih.gov/16210318/; https://pubmed.ncbi.nlm.nih.gov/19638342/; https://pubmed.ncbi.nlm.nih.gov/22344175/). Enzyme assays were typically conducted in technical duplicates or triplicates and repeated on at least one independent preparation of enzyme or cell lysates. Biological duplicates or triplicates were used in the lipid analyses of bacterial strains and key LC/MS analyses were performed in technical duplicates as described below. Methods followed the Rigor and Reproducibility requirements determined in NIH/NIAID grants # AI064798 and AI155674 that supported this work.
Data exclusions	No data were excluded from the analyses.
Replication	Enzyme assays using purified enzymes or cell lysates were typically conducted in technical duplicate or triplicate as indicated in the figure legends and/or Methods section and repeated at least two times using independent enzyme preparations or cell lysates (biological replicates). Total lipid analyses were conducted at least two times on independent bacterial cultures and were sometimes repeated on the same bacterial strains cultured under different conditions (e.g., on agar plates or in liquid medium). Throughout the figures, the results of one representative experiment are shown. LC/MS analyses were typically performed in technical duplicate. In some cases (e.g., Msmg plsM knock-down complemented with plsC from E. coli), independent recombinant clones were analyzed to ascertain that phenotypic changes observed in these strains followed the same trends independent of any potential genetic drift as these strains were being subcultured.
Randomization	In the study, experiments were done pair-wise with recombinant strains or cell lysates derived thereof being directly compared (for growth rate, fatty acid or lipid composition, enzyme activity) to control parent strains/lysates expressing normal levels of the wild-type gene. Therefore, no distribution in study groups was performed.
Blinding	Investigators were not blinded to data collection which required direct culturing of recombinant and control strains or addition of the proper enzymes (PlsM, PlsB2 or no enzyme) or cell lysates to the reaction mixtures. Investigator blinding was performed for analysis of LC/MS and GC/MS data.

Reporting for specific materials, systems and methods

We require information from authors about some types of materials, experimental systems and methods used in many studies. Here, indicate whether each material, system or method listed is relevant to your study. If you are not sure if a list item applies to your research, read the appropriate section before selecting a response.

Materials & experimental systems

n/a	Involved in the study
☒	☐ Antibodies
☒	☐ Eukaryotic cell lines
☒	☐ Palaeontology and archaeology
☒	☐ Animals and other organisms
☒	☐ Clinical data
☒	☐ Dual use research of concern

Methods

n/a	Involved in the study
☒	☐ ChIP-seq
☒	☐ Flow cytometry
☒	☐ MRI-based neuroimaging
